# Emergent Minimally Invasive Esophagogastrectomy

**DOI:** 10.1155/2017/4308628

**Published:** 2017-03-29

**Authors:** Thomas Fabian, Dorothy Chiaravalle, Jeremiah Martin

**Affiliations:** Department of Thoracic Surgery, Albany Medical Center, 50 New Scotland, Albany, NY 12208, USA

## Abstract

*Introduction*. Esophageal perforation in the setting of a malignancy carries a high morbidity and mortality. We describe our management of such a patient using minimally invasive approach.* Methods*. An 83-year-old female presented with an iatrogenic esophageal perforation during the workup of dysphagia. She was referred for surgical evaluation immediately after the event which occurred in the endoscopy suite. Minimally invasive esophagectomy was chosen to provide definitive treatment for both her malignancy and esophageal perforation.* Results*. Following an uncomplicated operative course, she was eventually discharged to extended care for rehabilitation and remains alive four years after her resection.* Conclusion*. Although traditional open techniques are the accepted gold standard of treatment for esophageal perforation, minimally invasive esophagectomy plays an important role in experienced hands and may be offered to such patients.

## 1. Case Report

We present the case of an 83-year-old Caucasian female who presented to our institution with a one-month history of abdominal pain, dysphagia, and weight loss. She was admitted to the medical service for inpatient evaluation of her symptoms. Her medical history was significant for depression, lifelong blindness, chronic back pain, cholelithiasis, and a right-sided radical mastectomy.

Upper GI endoscopy revealed an exophytic, ulcerating, obstructing mass at 30 cm from the incisors. The stricture was traversed with some difficulty and was noted to end at the gastroesophageal junction. At this point, attempt was made to pass a lubricated nasogastric tube beyond the stricture under direct vision. However it became apparent that there was now an extraluminal tract. Postprocedure computed tomography (CT) scan confirmed the suspected diagnosis of esophageal perforation of the intrathoracic esophagus.

Thoracic surgery was consulted and it was elected to perform a minimally invasive esophagectomy given the short interval of time between perforation and definitive surgical treatment. Esophagogastrectomy was performed via a right chest (thoracoscopic), abdomen (laparoscopic), and cervical anastomosis. A feeding jejunostomy was placed with botulinum toxin injection of the pylorus. The procedure was uneventful with moderate contamination of the right pleural space which was irrigated until clean.

Postoperatively the patient recovered well with no immediate complications from the surgery. Histopathology confirmed a diagnosis of poorly differentiated adenocarcinoma of the distal esophagus and stomach. Following surgery she suffered from altered mental status secondary to hyperammonemia and required multiple recurrent intubations for hypercarbia. She underwent tracheostomy and when her obtunded state resolved no longer required any ventilatory support. She suffered no other complications, required no additional interventions, and developed no infections. She returned to her preoperative living situation at an extended care facility where she resumed her normal activities. She remains well now four years after her esophagectomy.

## 2. Discussion

Esophageal perforation following instrumentation is an uncommon but important complication and one which retains a high morbidity and mortality [1]. The causes of perforation vary and the optimal treatment of this condition depending on the time and nature of presentation remains a topic of discussion [2]. It has been suggested that definitive treatment within 24 hours of perforation is associated with mortality of 10% to 25%; however this rate increases to 40% to 60% if treatment is delayed up to 48 hours [3]. In addition, it is generally accepted that perforation in the setting of a malignant stricture is best treated with simultaneous and definitive management of both the perforation and the malignant process. In the case of a malignant esophageal process with perforation following instrumentation, this generally requires esophagectomy [4, 5].

Early diagnosis is essential and to this end, a high index of suspicion must be maintained when managing a bulky esophageal mass. As there are treatment options, there is some debate surrounding the optimum choice of imaging modality. The usual initial test is a contrast esophagram; however such studies may yield false negative results in 10% of cases [6]. Water-soluble contrast agents have been shown to identify only 50% of cervical perforations and 80% of esophageal perforations [7]. In the setting of a negative esophagram, a CT scan should be performed which may indicate evidence of mediastinal air or abscess formation [6].

Once the perforation has been identified, the choice of operative approach must be considered. The use of esophagectomy for perforation was initially reported in the 1960s [8]. At present, esophageal resection is the preferred treatment of a perforated malignant stricture. Cervical anastomosis allows for safe drainage without further contamination of the mediastinum in the event of an anastomotic leak. Traditional approaches include the use of a laparotomy with or without thoracotomy and a lateral neck dissection for creation of a cervical anastomosis.

The role of minimally invasive surgery continues to expand, and since its introduction there has been concern as to whether or not it can be applied in the setting of perforation and contamination [9]. Traditional teaching held that the rate of complications including abscess formation and wound infection would be increased with laparoscopic techniques due to the technical difficulties presented by the case. That has not been the case, and it is now recognized that laparoscopy in the setting of perforated appendicitis is associated with shorter length of stay and indeed decreased need for antibiotics and incidence of wound infection [10].

Minimally invasive esophagectomy (MIE) has emerged as a means to reduce the morbidity and mortality of esophagectomy; however there is still little documentation of treatment outcomes in the setting of acute perforation. In 2002 Landen and El Nakadi [11] reported a pilot series of minimally invasive repair in the setting of Boerhaave's syndrome with acceptable outcomes. Gupta [12] reported their transhiatal approach for distal perforations in the setting of malignancy. We have previously reported our experience with the widespread application of minimally invasive approaches to our esophagectomy practice and discussed some of the technical advantages that the procedure affords [13–15]. MIE offers the advantages of thoracoscopic visualization without the morbidity of a thoracotomy. This may potentially be of great benefit to the elderly [16] and may reduce the physiologic strain on an already critically ill patient with esophageal perforation.

MIE is gaining recognition as a procedure which offers results equal to open procedures with lessened morbidity at centers experienced in the approach [17, 18]. With the high morbidity and mortality associated with esophageal perforation, MIE stands as one means by which we might improve outcomes in these patients. Further study is required to determine if MIE is as effective and safe in the setting of a perforated malignancy as traditional open procedures.
